# Iridoids rich fraction from *Valeriana jatamansi* Jones promotes axonal regeneration and motor functional recovery after spinal cord injury through activation of the PI3K/Akt signaling pathway

**DOI:** 10.3389/fnmol.2024.1400927

**Published:** 2024-05-02

**Authors:** Yunyun Wang, Jiachun Lu, Hua Xiao, Lijuan Ding, Yongzhi He, Cong Chang, Wenchun Wang

**Affiliations:** ^1^Department of Rehabilitation Medicine, The General Hospital of Western Theater Command, Affiliated Hospital of Southwest Jiaotong University, Chengdu, Sichuan, China; ^2^Chengdu Eighth People’s Hospital (Geriatric Hospital of Chengdu Medical College), Chengdu, Sichuan, China; ^3^North Sichuan Medical College, Chengdu, Sichuan, China

**Keywords:** Iridoid-rich fraction derived from *Valeriana jatamansi* Jones, spinal cord injury, axonal regeneration, PI3K/Akt signaling, traditional Chinese medicine

## Abstract

*Valeriana jatamansi* Jones (VJJ), renowned for its extensive history in traditional Chinese medicine and ethnomedicine within China, is prevalently utilized to alleviate ailments such as epigastric distension and pain, gastrointestinal disturbances including food accumulation, diarrhea, and dysentery, as well as insomnia and other diseases. Moreover, the Iridoid-rich fraction derived from *Valeriana jatamansi* Jones (IRFV) has demonstrated efficacy in facilitating the recuperation of motor functions after spinal cord injury (SCI). This study is aimed to investigate the therapeutic effect of IRFV on SCI and its underlying mechanism. Initially, a rat model of SCI was developed to assess the impact of IRFV on axonal regeneration. Subsequently, employing the PC12 cell model of oxidative damage, the role and mechanism of IRFV in enhancing axonal regeneration were explored using the phosphoinositide-3-kinase (PI3K)/protein kinase B (Akt) signaling pathway inhibitor LY294002. Ultimately, the same inhibitor was administered to SCI rats to confirm the molecular mechanism through which IRFV promotes axonal regeneration by activating the PI3K/Akt signaling pathway. The results showed that IRFV significantly enhanced motor function recovery, reduced pathological injury, and facilitated axonal regeneration in SCI rats. *In vitro* experiments revealed that IRFV improved PC12 cell viability, augmented axonal regeneration, and activated the PI3K/Akt signaling pathway. Notably, the inhibition of this pathway negated the therapeutic benefits of IRFV in SCI rats. In conclusion, IRFV promote promotes axonal regeneration and recovery of motor function after SCI through activation of the PI3K/Akt signaling pathway.

## 1 Introduction

Spinal cord injury (SCI) represents a critical traumatic condition frequently culminating in profound motor and sensory dysfunctions (Zhou et al., 2023). The etiology of SCI encompasses high-impact traumas including traffic collisions, falls, and violent incidents, in addition to infections, neoplasms, degenerative spinal pathologies, ischemia–reperfusion events, and vascular factors (Hu X. et al., 2023; Hu Z. et al., 2023). Over the decade spanning January 1, 2011, to December 31, 2020, SCI incidence was recorded at 26.5 cases per million individuals, predominantly characterized by cervical injuries (52.1%). The leading cause of these injuries was identified as traffic-related incidents (29.9%), closely followed by occupational hazards (29.8%) (Barbiellini et al., 2022). The formidable challenges in SCI repair lie in its intricate pathomechanisms and the inherent difficulty of nerve regeneration within the central nervous system. Consequently, identifying effective strategies for axonal regeneration and neural circuitry remodeling is a pivotal therapeutic concept in SCI management (Barbiellini et al., 2022).

Revered as a “bright pearl” in the global “medical crown,” Traditional Chinese Medicine (TCM) boasts a millennia-long history of clinical application. The integration of TCM as an adjunctive therapy for spinal cord injury (SCI) has garnered escalating attention from international researchers, heralding a new epoch in SCI treatment (Lu et al., 2020). Zhizhuxiang, a prized herbal remedy, is derived from the dried root and rhizome of Valeriana jatamansi Jones (VJJ) (Zhao et al., 2022), first documented in the Compendium Materia Medica. Renowned for its analgesic, antidiarrheal, and sedative properties, it holds a venerable position in herbal medicine (Wang et al., 2018). Our preceding research revealed that the Iridoid-rich fraction from Valeriana jatamansi Jones (IRFV) significantly enhances motor function recovery following SCI in rat models, reduce inflammatory response, and promote the expression of brain-derived neurotrophic factor (BDNF) and nerve growth factor (NGF), with the optimal efficacy noted at a dosage of 10 mg/kg (Xiong et al., 2022).

In our prior investigation, we retrieved the principal constituents of VJJ via literature data mining and applied network pharmacology alongside molecular docking methodologies to forecast and corroborate VJJ’s molecular mechanism in SCI treatment. Our findings indicate that VJJ might exert its therapeutic effects in SCI primarily through iridoids, including baldrinal, valtrate, jatamanin C, isovaltrate, deacetylisovaltrate, and valeriandoid F, with the mechanism potentially associated with the phosphoinositide-3-kinase (PI3K)/protein kinase B (Akt) signaling pathway.

The PI3K/Akt signaling pathway, a multifaceted network involving a plethora of regulators and effectors, exerts a critical regulatory influence on diverse physiological functions within the central nervous system (CNS), encompassing the regulation of neuronal cell growth, survival, and axonal development. The PI3K/Akt signaling cascade is pivotal in the pathological progression of SCI (Xiao et al., 2022), with its activation markedly facilitating the process of axonal regeneration post-SCI (Hausott et al., 2022).

In this study, we employed a rat model of SCI to ascertain whether the IRFV can enhance motor function recovery and facilitate axonal regeneration post-SCI. Additionally, we utilized the PC12 cell oxidative damage model to further explore its underlying mechanism.

## 2 Method

### 2.1 Extraction and preparation of IRFV

VJJ was purchased from Hebei Renxin Pharmaceutical Co., LTD. The extraction process was as follows: the crude powder of VJJ was subjected to 70% ethanol extraction, with an initial extraction ratio of 8:1 for 24 h, followed by a subsequent 6:1 extraction for 12 h. The resultant extracts were amalgamated and then concentrated under reduced pressure to remove any residual alcoholic odor, yielding an ethanolic extract. For purification, the ethanolic extract was homogeneously dispersed in water through ultrasonication and subsequently loaded onto a D101 macroporous resin column, characterized by a specific adsorption capacity of 72 mg/g, an adsorption flow rate of 1 BV/h, and an elution flow rate of 2 BV/h. Elution was performed sequentially with water at 6BV, followed by 60% ethanol at 4BV, and finally 95% ethanol at 4BV. The 95% ethanol fraction was collected, concentrated into a paste under reduced pressure, and subsequently dried in a vacuum at 40°C. The quantification of total iridoids in the extract, as determined by ultraviolet spectrophotometry, was established at 83.25%.

### 2.2 Animals and SCI model

Forty-eight healthy male Sprague Dawley rats, weighing between 250 to 290 grams, were acquired from SiPeiFu (Beijing) Biotechnology Co. Ltd. (Animal Certificate No.: SCXK (Beijing) 2019–0010). The housing conditions were meticulously maintained, ensuring cleanliness and adequate ventilation, with a stable room temperature of 22 ± 2°C, relative air humidity maintained at 60%, a 12-h light–dark cycle, and *ad libitum* access to food. The conduct of the animal study received ethical approval from the Ethics Committee of the General Hospital of the Western Theater Command of the Chinese People’s Liberation Army.

Anesthesia induction in the rats was achieved through intraperitoneal injection of 5% pentobarbital sodium, administered at a dosage of 30 mg/kg. Subsequent to the T10 laminectomy, the spinal cord was clamped at the corresponding level for 10 s using 70 g medical aneurysm clips. Following the closure of the surgical incision, penicillin was administered via intramuscular injection at a dosage of 200,000 U/day for a period of 3 days. Additionally, manual bladder expression was conducted thrice daily to facilitate urinary excretion until normal voiding was reestablished. In the sham group, the procedure was limited to laminectomy, ensuring the spinal cord was left intact.

Forty-eight rats were randomly assigned into four groups using the random number table method: Sham, SCI, IRFV, and IRFV+LY294002. The IRFV extract, weighing 0.5 g, was dissolved via ultrasonic agitation in a 0.5% carboxymethyl cellulose sodium (CMC-Na) solution, which was subsequently brought to a final volume of 500 mL. The IRFV group received the IRFV solution orally at a dose of 10 mg/(kg·d). For the IRFV+LY294002 group, in addition to the same oral dose of IRFV, an intraperitoneal injection of LY294002 was administered at 1 mg/(kg·d). The Sham and SCI groups were administered an equivalent volume of CMC-Na solution daily via gavage. The duration of this intervention extended over a period of 28 days.

### 2.3 Behavioral testing

For the assessment of hind limb motor function in rats, the Basso, Beattie, and Bresnahan scoring (BBB score) (Basso et al., 1995) was employed. Two independent experimenters, well-versed in the scoring criteria and blinded to the animal groupings, conducted the evaluations. Each limb was scored separately over a duration of 5 min, and the average of the two evaluators’ scores was recorded for each assessment.

### 2.4 Luxol fast blue staining and Nissl staining

Spinal cord tissues were fixed in a 10% neutral buffered formalin solution for 24 h. Following fixation, the tissues underwent standard dehydration and embedding procedures, after which 4 μm serial sections were prepared for Luxol Fast Blue (LFB) and Nissl staining. For LFB staining, sections were deparaffinized to 95% ethanol, followed by immersion in the fast blue staining solution (Solarbio, Beijing, China) overnight at room temperature, subsequently rinsed in 95% ethanol to remove excess dye, followed by distilled water and then differentiated in LFB Differentiation solution for 15 s, and further in 70% ethanol for 30 s until the demarcation between gray and white matter was distinct, followed by a distilled water rinse, counterstaining with Eosin for 2 min, and then rapid washing and routine dehydration, clarification, and sealing of the sections. In the Nissl staining process, following routine dewaxing, sections were immersed in toluidine blue staining solution (Solarbio, Beijing, China), maintained at 50–60°C for 20–40 min, Upon completion of the staining, the tissue sections were gently rinsed with distilled water, swiftly differentiated using 95% ethanol, and subsequently underwent standard dehydration, clarification, and sealing procedures.

### 2.5 Cell culture and drug treatment

Treatment Highly differentiated PC12 cells were purchased from Wuhan Pricella Life Science and Technology Co. PC12 cells were cultured in Dulbecco’s modified Eagle’s medium (DMEM, Gibco, United States) and maintained in an incubator at 37°C and 5% CO_2_. In the study, the cells were allocated into four experimental groups: Control, H_2_O_2_, H_2_O_2_ + IRFV, and H_2_O_2_ + IRFV+LY294002. Before commencing the experiment, PC12 cells were seeded in 96-well plates at a concentration of 2 × 10^4^ cells/mL. The control group was maintained in DMEM without additional treatments. The H_2_O_2_ group received 400 μmol/L H_2_O_2_ (dissolved in DMEM) solution for 2 h. The H_2_O_2_ + IRFV group was exposed to 400 μmol/L H_2_O_2_ solution for 2 h, followed by treatment with 10 μg/mL IRFV solution (dissolved in DMEM) for 24 h. The H_2_O_2_ + IRFV+LY294002 group underwent 400 μmol/L H_2_O_2_ exposure for 2 h, succeeded by a combined treatment (dissolved in DMEM) of 10 μg/mL IRFV and 10 μmol/L LY294002 for 24 h. Each group consisted of three replicate wells to ensure experimental validity.

### 2.6 Cell counting Kit-8 assay

Cell viability was assessed using the Cell Counting Kit-8 (CCK-8) assay. A range of H2O2 concentrations (100–800 μmol/L) and IRFV concentrations (0.1–20 μg/mL) were applied to cells in 96-well plates. The Cell Counting Kit-8 reagent (MedChemExpress, New Jersey, United States) was introduced to the 96-well plates and the plates were incubated for 1 h in a cell incubator. Subsequently, the optical density at 450 nm was determined using a spectrophotometer (ThermoFisher Scientific, United States). Each experiment was replicated three times for consistency. The formula for calculating cell viability was as follows: Cell viability (%) = [(Absorbance in drug-treated wells - Absorbance in blank wells) / (Absorbance in control wells - Absorbance in blank wells)] × 100%.

### 2.7 Immunofluorescence

Spinal cord tissues were fixed in a 10% neutral buffered formalin solution for 24 h. Following fixation, tissues underwent standard dehydration and embedding processes, were serially sectioned at 4 μm thickness, and the sections were subjected to antigen retrieval in EDTA solution (Solarbio, Beijing, China) using a microwave. PC12 cells were fixed using 4% paraformaldehyde for a duration of 20 min. Tissue and cell samples were permeabilized in Triton X-100 (Solarbio, Beijing, China) for 20 min, blocked with 3% BSA for 1 h at room temperature, and incubated overnight at 4°C with anti-neurofilament 200 (NF200) (1:200, 2,836, Cell Signalling Technology, Beverly, MA, United States) and growth-associated protein 43 (GAP43) (1:200, 8,945, Cell Signalling Technology, Beverly, MA, United States) primary antibodies. Subsequently, the samples were incubated with rabbit (1:200, Cat No: SA00013-1, Proteintech, Wuhan, China) or mouse (1:200, Cat No: SA00013-1, Proteintech, Wuhan, China) secondary antibodies conjugated with fluorochrome for 1 h, followed by re-staining of cell nuclei with DAPI for 1 h and sealing. Imaging was performed using a Nikon ECLIPSE CI-L microscope, and fluorescence intensity was quantitatively analyzed with Image J software.

### 2.8 Western blotting

The total protein from spinal cord tissues and cell samples was extracted using a protein extraction kit (Solarbio, Beijing, China), following the provided instructions, and the protein concentration was quantified using the BCA method. After gel preparation, the samples were loaded in equal amounts of total protein. The proteins was separated by 10% SDS-PAGE (80 V, 2 h, then 130 V, 1 h) and transferred to a PVDF membrane. The membrane was blocked with 5% milk for 1 h, followed by overnight incubation at 4°C with primary antibodies against NF200 (1:1000, 2,836, Cell Signalling Technology, Beverly, MA, United States), GAP43 (1:1000, 8,945, Cell Signalling Technology, Beverly, MA, United States), PI3K (1:1000, 4,292, Cell Signalling Technology, Beverly, MA, United States), p-PI3K (1:500, AF3242, Affinity, China), Akt (1:1000,bs-6951R, Bioss, Beijing, China), p-Akt (1:1000,bsm-60645R, Bioss, Beijing, China), and GAPDH (1:8000, 2,118, Cell Signalling Technology, Beverly, MA, United States). This was succeeded by incubation with HRP-conjugated secondary antibodies (1:10000, 7,074, 7,076, Cell Signalling Technology, Beverly, MA, United States) for 1 h at room temperature. Lastly, the membranes were visualized using ECL reagents and the Azure Biosystems NIR Fluorescence Imaging system, followed by quantitative grayscale analysis using Image J software.

### 2.9 Statistical analysis

Statistical analysis was performed using SPSS software version 25.0 (SPSS, Chicago, IL, United States). Comparative analysis among multiple groups was conducted using one-way ANOVA, while the differences between two groups were evaluated through unpaired Student’s t-test. A *p*-value of less than 0.05 was deemed to indicate statistical significance.

## 3 Result

### 3.1 IRFV facilitates tissue regeneration and motor functional recovery post-SCI

**Figure 1:**
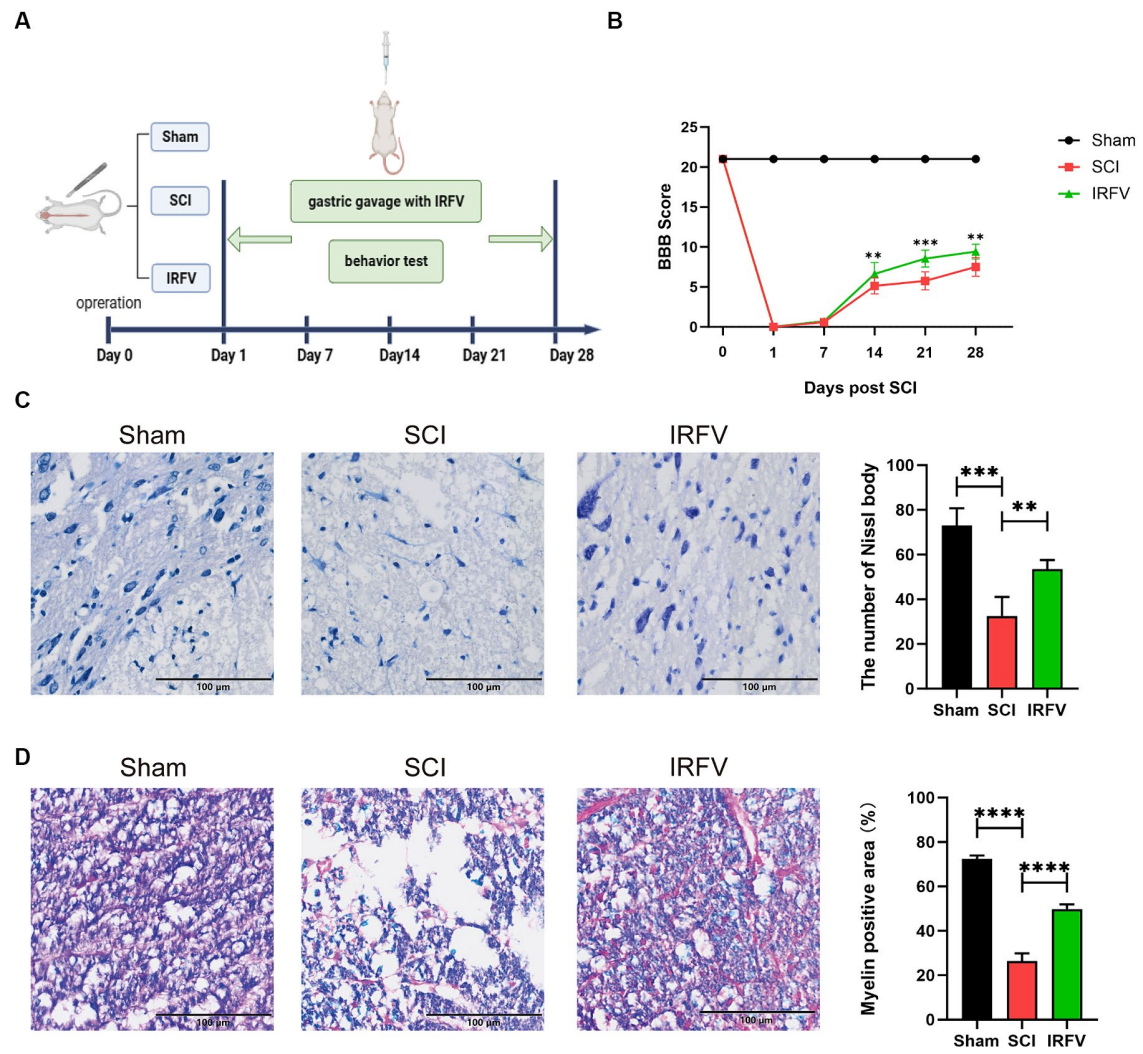
IRFV facilitates tissue regeneration and motor functional recovery post-SCI. **(A)** Establishmen of rat SCI model and IRFV treatment protocol. **(B)** BBB scores of rats up to 28 days after operation. **(C)** Number of Nissl bodies in rat spinal cord tissues analyzed by Nissl staining to analyze neuronal survival. **(D)** Myelin positive area in rat spinal cord tissues analyzed by LFB staining to analyze loss of myellin. Scale bar = 100 μm. Data are presented as mean ± SD. ^*^p < 0.05, ^**^p < 0.01, ^***^p < 0.001.

IRFV was administered via gastric gavage at a consistent dosage of 10 mg/kg daily for 28 days following SCI. Postoperatively, motor function was evaluated on days 0, 7, 14, 21, and 28 post-SCI using the BBB score (Figure 1A). The results indicate that on the first day post-operation, the Sham group had a BBB score of 21, while all other groups scored 0, suggesting the model was successfully established. The results also indicated that no significant difference in BBB scores between the SCI and IRFV groups from day 0 to 7. However, from day 14 to 28 post-operation, IRFV treatment significantly enhanced BBB scores, consistently outperforming the SCI group (Figure 1B). Histological analysis of spinal cord tissues using Nissl staining revealed a marked reduction in Nissl bodies in the SCI group compared to the sham group. Conversely, IRFV treatment notably increased the number of Nissl bodies compared to the SCI group (Figure 1C). Myelin loss in tissue was assessed using LFB staining, demonstrating that IRFV treatment significantly mitigated myelin loss compared to the SCI group (Figure 1D). In conclusion, these findings indicate that IRFV plays a pivotal role in facilitating tissue repair and remyelination, thus enhancing functional recovery post-SCI *in vivo*.

### 3.2 IRFV promotes axonal regeneration after SCI, potentially through the activation of the PI3K/Akt signaling pathway

**Figure 2:**
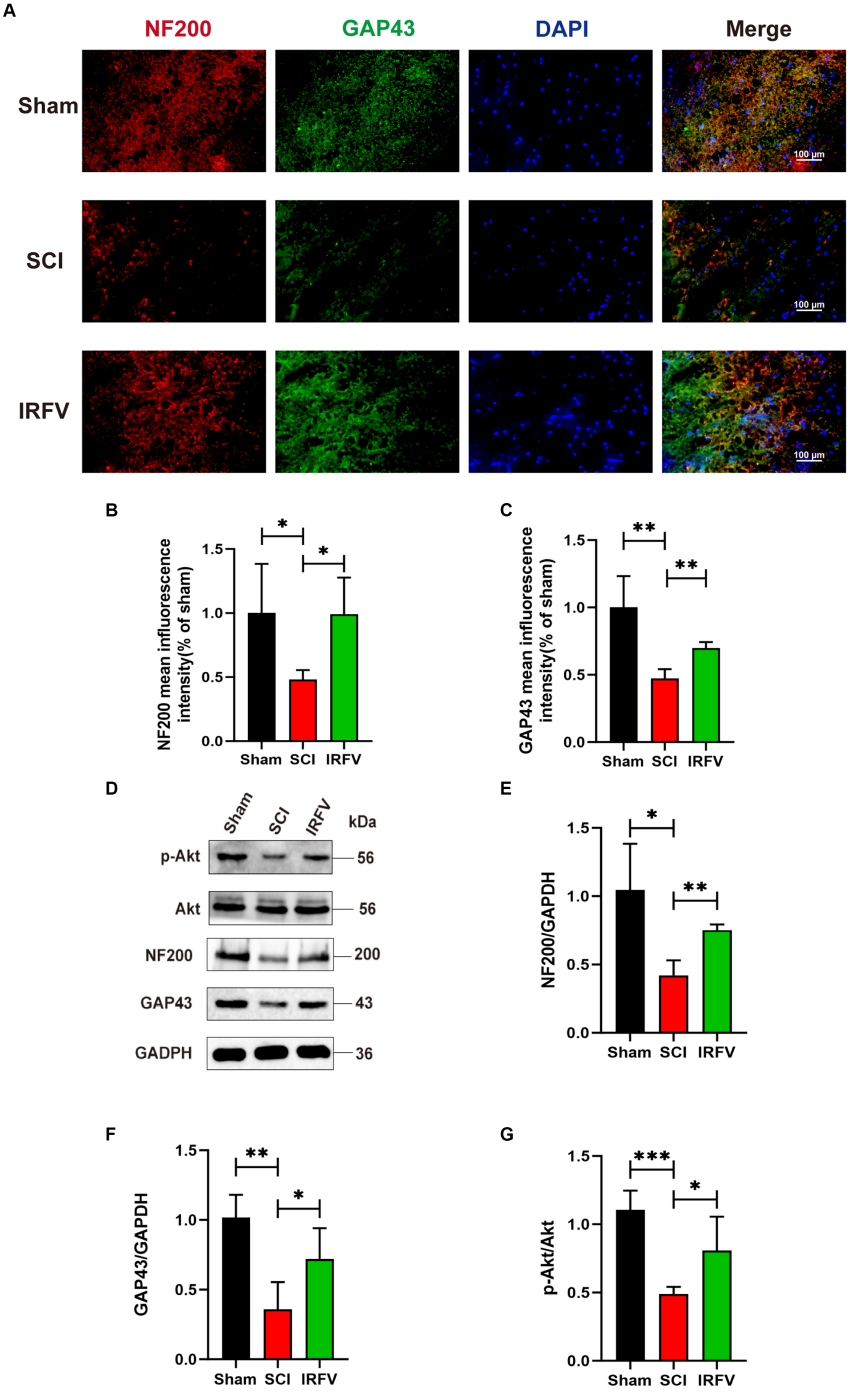
IRFV promotes axonal regeneration after SCI, potentially through the activation of the PI3K/Akt signaling pathway. **(A–C)** The expression of NF200 (red) and GAP43 (green) was analyzed by immunofluorescence. Nuclear chromatin was stained with DAPI (blue). Scale bar = 100 μm. **(D–G)** Western blotting analysis of NF200, GAP43, Akt and p-Akt expression in spinal cord tissue. ^*^p < 0.05, ^**^p < 0.01, ^***^p < 0.001.

NF200, a crucial neurofilament predominantly located in the cytoplasm of neurons and axons, serves as an indirect indicator of the extent of axonal damage and regeneration following SCI (Hu X. et al., 2023; Hu Z. et al., 2023). GAP43 is an axonal membrane protein that plays a role in axonal development, regeneration and plasticity (Liu et al., 2020). Immunofluorescence co-localization and western blotting echniques were utilized to assess the expression of axon-related proteins NF200 and GAP43 in various groups of rats, four weeks post-SCI. Immunofluorescence co-localization results revealed a significant reduction in the expression of NF200 (*p* < 0.05) and GAP43 (*p* < 0.01) in the SCI group compared to the Sham group, while IRFV treatment markedly enhanced the expression levels of NF200 (*p* < 0.05) and GAP43 (*p* < 0.01) (Figures 2A–C). This result was further corroborated by Western blotting. Moreover, western blotting analysis demonstrated that IRFV treatment elevated the protein expression of p-Akt (*p* < 0.05), suggesting a potential link between IRFV’s axon regeneration-promoting effect in SCI and Akt phosphorylation (Figures 2D–G).

### 3.3 IRFV improves cell viabilty of PC12 cells

**Figure 3:**
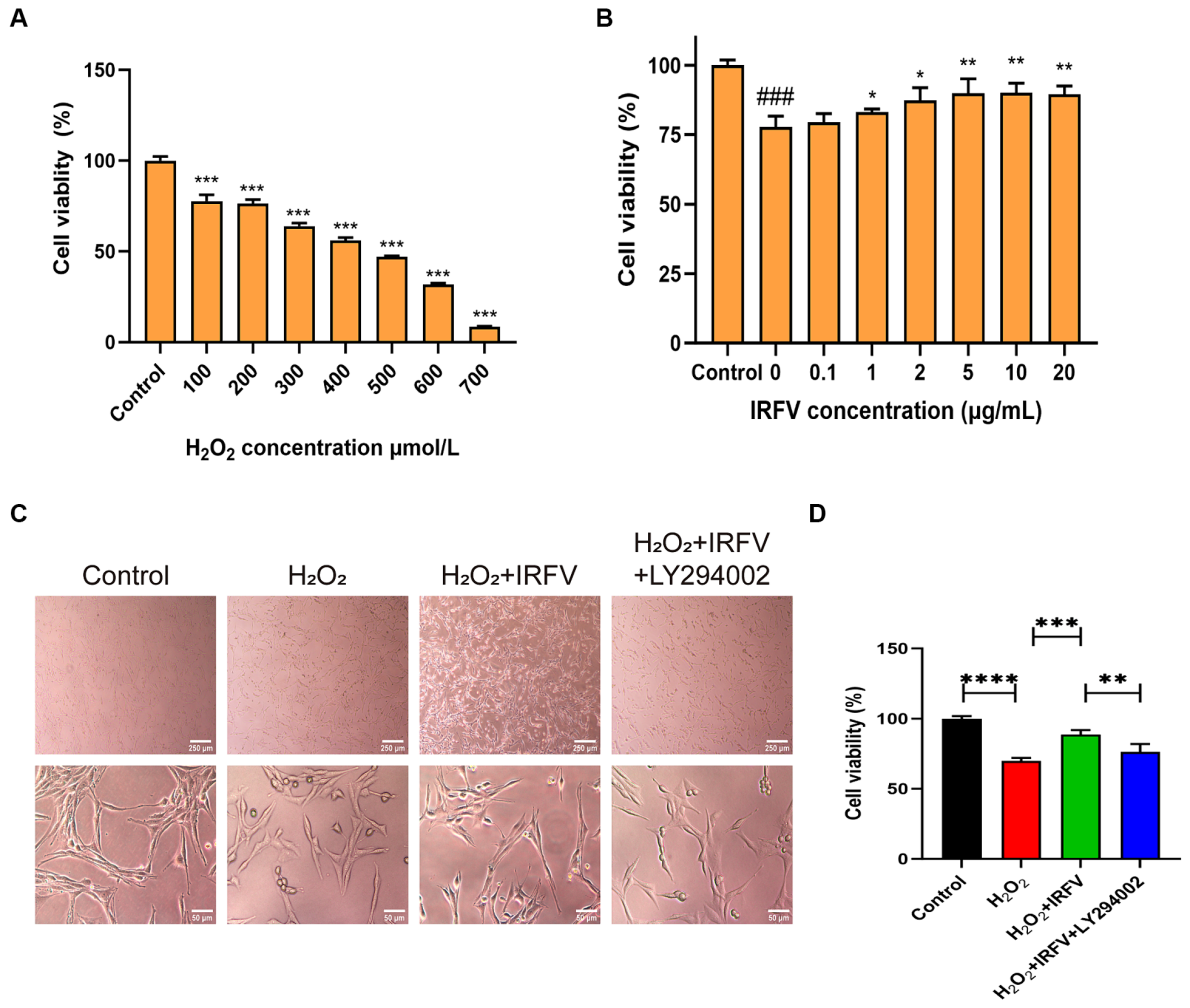
IRFV improves cell viabilty of PC12 cells. **(A)** Cell viability of PC12 cells with oxidative damage after treating with H_2_O_2_ at different concentrations for 2 h. **(B)** Cell viability of PC12 cells treated with different concentrations of IRFV after treating with 400 μmol/L H_2_O_2_ for 2 h. **(C)** Cell morphology of PC12 cells. Scale bar = 250 μm, 50 μm **(D)** Cell viability of PC12 in four groups. Data are presented as mean ± SD. ^*^p < 0.05, ^**^p < 0.01, ^***^p < 0.001.

The CCK-8 assay was employed to determine the optimal intervention concentration of H_2_O_2_ and IRFV. Results indicated that the viability of PC12 cells was approximately 50% following 400 μmol/L H_2_O_2_ treatment for 2 h (Figure 3A). Consequently, 400 μmol/L was chosen as the H_2_O_2_ treatment concentration. Following 2 h of H_2_O_2_ treatment, PC12 cells treated with 10 μg/mL IRFV for 24 h exhibited the most significant increase in viability compared to other concentrations of IRFV administration (Figure 3B), thus 10 μg/mL was selected as the administration concentration in this study.

Optical microscopy revealed that the morphology of PC12 cells in the Control group was pike-shaped, featuring large cytosol, thickened and elongated cytosolic branches and trunks, strong refractive index, extended pseudopods, and a high cell count. Compared with the Control cells, the synapses of the PC12 cells in the H_2_O_2_ group and the H_2_O_2_ + IRFV+LY294002 group were observed to be shorter or the pseudopods absent. Additionally, a portion of these cells crumpled, rounded, and detached; the intercellular gap widened, and there was a noticeable reduction in cell count. In the H_2_O_2_ + IRFV group, there was a notable extension of pseudopods, reduction in cell gap, increase in cell number, and a clear improvement in the growth status of the cells (Figure 3C). These findings imply that IRFV contributes to the enhancement of PC12 cell growth, and that this effect can be reversed by the inhibition of PI3K/Akt through LY294002. The CCK-8 assay results demonstrated a significant reduction in the viability of the PC12 cells in the H_2_O_2_ group compared to the Control group (*p* < 0.001). Conversely, cell viablity in the H_2_O_2_ + IRFV group was significantly elevated compared with that of H_2_O_2_ group (*p* < 0.01), while the H_2_O_2_ + IRFV+LY294002 group exhibited a marked decrease in cell viability compared to the H_2_O_2_ + IRFV group (*p* < 0.01) (Figure 3D). This suggests that IRFV improves the cell viability, an effect that can be reversed by the inhibition of PI3K/Akt via LY294002.

### 3.4 IRFV facilitates axonal regeneration after H_2_O_2_ treatment in PC12 cells

**Figure 4:**
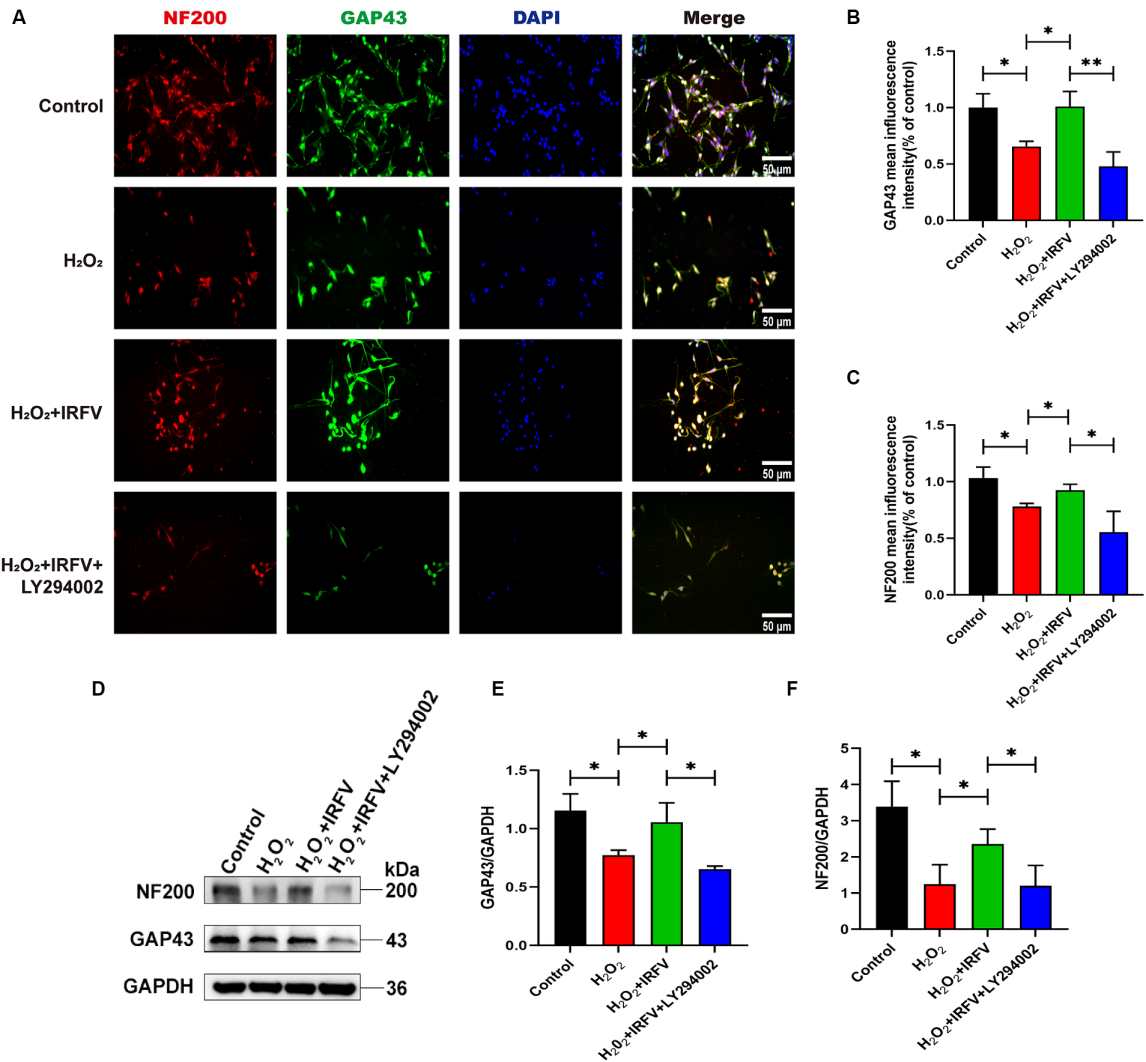
IRFV facilitates axonal regeneration after H_2_O_2_ treatment in PC12 cells. **(A–C)** The expression of NF200 (red) and GAP43 (green) was analyzed by immunofluorescence. Nuclear chromatin was stained with DAPI (blue). Scale bar = 50 μm. **(D–F)** Western blotting analysis of NF200 and GAP43 expression in PC12 cells. Data are presented as mean ± SD. ^*^p < 0.05, ^**^p < 0.01, ^***^p < 0.001.

Immunofluorescence and western blotting were employed\d to assess axon-related proteins NF200 and GAP43 in PC12 cells. Immunofluorescence results revealed a significant decrease in the protein expression of NF200 and GAP43 in the H_2_O_2_ group compared to the Control group (*p* < 0.05). The protein expression levels of NF200 and GAP43 in the H_2_O_2_ + IRFV group were significantly elevated compared to those in the H_2_O_2_ group (*p* < 0.05). Protein expression levels of NF200 (*p* < 0.01) and GAP43 (*p* < 0.05) in the H_2_O_2_ + IRFV+LY294002 were significantly decreased in comparison to the H_2_O_2_ + IRFV group (Figures 4A–C), which was further verified by western blotting results (Figures 4D–F). These findings indicate that IRFV facilitates axon regeneration following PC12 cell injury, and that this effect can be reversed by inhibiting the PI3K/Akt signaling pathway through the application of LY294002.

### 3.5 IRFV promotes axonal regeneration after H_2_O_2_ treatment via PI3K/Akt signalling

**Figure 5:**
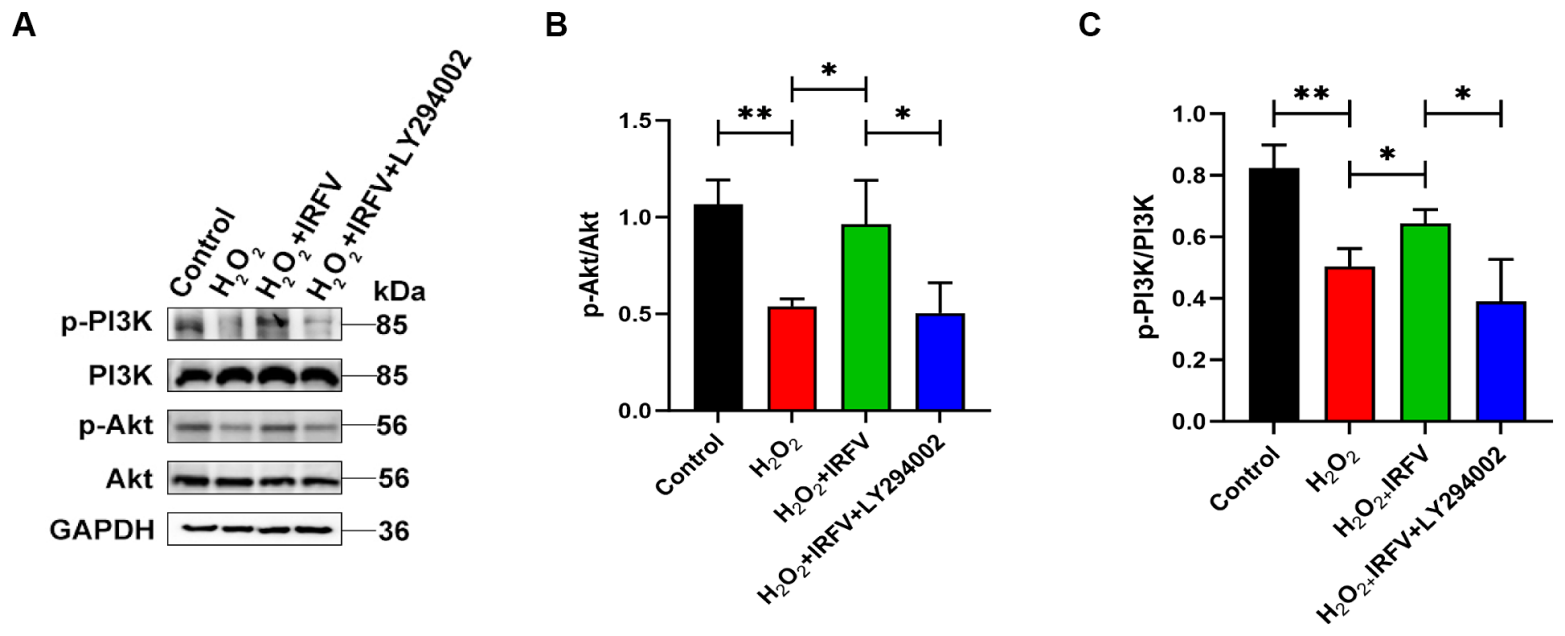
IRFV promotes axonal regeneration after H_2_O_2_ treatment via PI3K/Akt signalling. **(A–C)** Western blotting analysis of p-PI3K, PI3K, p-Akt, and Akt expression in PC12 cells. Data are presented as mean ± SD. ^*^p < 0.05, ^**^p < 0.01, ^***^p < 0.001.

Western blotting was utilized to detect the expression of PI3K/Akt pathway proteins, aiming to elucidate the potential mechanism underlying the promotion of axonal regeneration. The expression levels of PI3K/Akt protein were significantly upregulated in IRFV-treated, H_2_O_2_-pre-stimulated PC12 cells (*p* < 0.05), while a notable reduction was observed in the expression of PI3K/Akt protein following the application of LY294002 (*p* < 0.05), indicating effective inhibition of the PI3K/Akt signaling pathway (Figures 5A–C). These results imply that IRFV may promote axonal regeneration in PC12 cells by activating the PI3K/Akt signaling pathway.

### 3.6 Inhibition of the PI3K/Akt signalling reverses the treatment of IRFV after SCI

**Figure 6:**
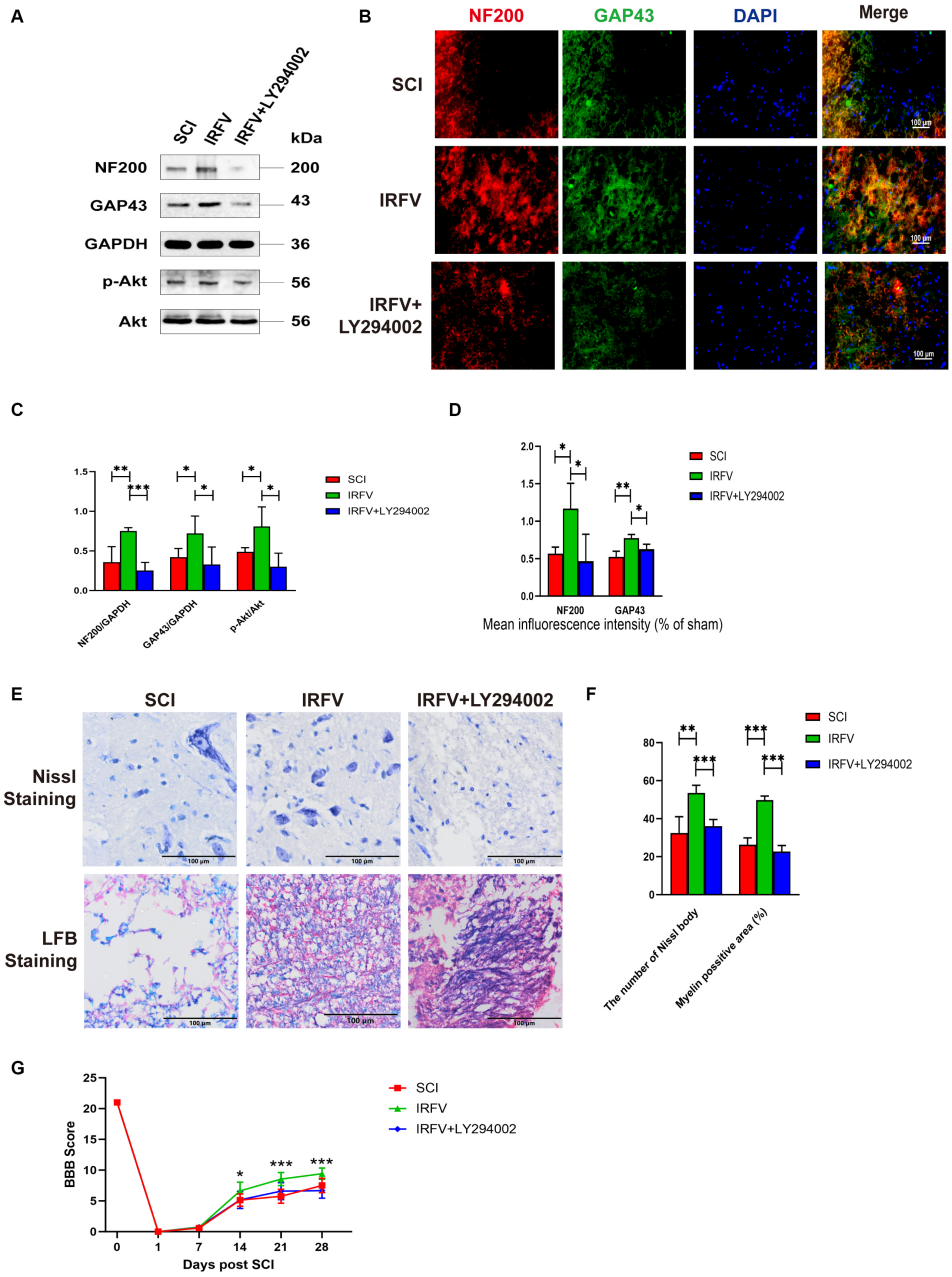
Inhibition of the PI3K/Akt signalling reverses the treatment of IRFV after SCI. **(A,C)** Western blotting analysis of NF200, GAP43, p-Akt, and Akt expression in spinal cord tissue of rats in SCI, IRFV and IRFV+LY294002 group. **(B,D)** Immunofluorescence analysis of NF200 (red) and GAP43 (green) expression in spinal cord tissue of rats in SCI, IRFV and IRFV+LY294002 group. Nuclear chromatin was stained with DAPI (blue). Scale bar = 100 μm. **(E,F)** Number of Nissl bodies in rat spinal cord tissues analyzed by Nissl staining to analyze neuronal survival, Myelin positive area in rat spinal cord tissues analyzed by LFB staining to analyze loss of myellin. Scale bar = 100 μm. **(G)** BBB scores of rats in SCI, IRFV and IRFV+LY294002 group. Data are presented as mean ± SD. ^*^p < 0.05, ^**^p < 0.01, ^***^p < 0.001.

Rats with SCI treated with IRFV were administered LY294002 via intraperitoneal injection. Western blot analysis of PI3K/Akt signaling pathway proteins in the rat spinal cord revealed that post-IRFV treatment, the expression of these proteins was significantly upregulated (*p* < 0.05), indicating activation of PI3K/Akt signaling pathway. After the intraperitoneal injection of LY294002, a significant decrease in the expression of PI3K/Akt signaling pathway proteins was observed in the rat spinal cord, suggesting effective inhibition of the PI3K/Akt pathway (Figures 6A,C–D). Immunofluorescence and Western blot analyses demonstrated that IRFV treatment significantly elevated the protein expression of NF200 and GAP43 in the spinal cord of the SCI rats, whereas LY294002 administration via intraperitoneal injection markedly reduced their expression (Figures 6A–D). Nissl and LFB staining results indicated that intraperitoneal administration of LY294002 reversed IRFV’s effects on increasing the number of Nissl bodies (*p* < 0.001) and ameliorating demyelination (*p* < 0.001) (Figures 6E,F). Behavioral assessments also revealed that intraperitoneal administration of LY294002 led to a significant reduction in motor function compared to IRFV-treated rats (Figure 6G). These findings demonstrate that the inhibition of the PI3K/Akt signaling pathway reverses IRFV’s effect on promoting axonal regeneration in SCI rats, further suggesting that IRFV promotes axonal regeneration by activating the PI3K/Akt pathway.

## 4 Discussion

SCI constitutes a complex tissue injury, resulting in permanent and degenerative damage to the CNS. Following SCI, deleterious cellular processes ensue, including axonal degeneration, neuronal loss, neuroinflammation, reactive gliosis, and scar formation (Clifford et al., 2023). The inability of the adult mammalian CNS to regenerate post-injury is the direct cause of the permanent loss of sensory and motor functions following SCI (Noristani, 2022). Axons, as structural and functional units of neural circuits, play a central role in neural signaling. Consequently, damage to either the CNS or the peripheral nervous system necessitates axonal regeneration for the restoration of neural function (Zhang et al., 2020). To date, one of the most promising pre-clinical therapeutic strategies has been neural stem cell therapy for SCI (de Freria et al., 2021). However, the application of neural stem cell transplantation is still limited due to the high price and complex drug delivery methods, as well as the potential adverse effects, including possible overgrowth or tumor formation and transplantation-induced pain. VJJ represents a category of medicinal plant resources exhibiting exceptional developmental potential within the domain of TCM. Furthermore, it is devoid of toxicological side effects and evident drug dependence (Yu et al., 2015). Our previous research has established that IRFV can enhance motor function recovery and elevate the expression levels of brain-derived neurotrophic factor and nerve growth factor in SCI rats (Xiong et al., 2022). In this study, aligning with prior findings, our results corroborate that IRFV enhances motor function in SCI rats. Furthermore, Nissl staining results verified that IRFV treatment led to an increase in the number of Nissl bodies within the spinal cord tissues of SCI rats, while LFB staining results substantiated that IRFV treatment ameliorated demyelination in SCI rats. Western blotting and immunofluorescence analyses demonstrated that IRFV treatment elevated the expression of axon-related proteins NF200 and GAP43. Collectively, these findings suggest that IRFV facilitates motor function recovery in SCI rats by promoting axonal regeneration.

PTEN is widely recognized as a crucial intrinsic inhibitor of CNS axon regeneration. The PI3K/AKT/mTOR pathway, under the negative regulation of PTEN, is pivotal in diverse models of axon injury (Zhang et al., 2018). The PI3K/AKT pathway represents a vital survival mechanism, orchestrating signaling, differentiation, and axon growth. Activated AKT notably diminishes Fas ligand expression by suppressing the pro-apoptotic transcription factors Bad and Forkhead (Akram et al., 2022). On the other hand, phosphorylated AKT promotes cytoskeletal filament assembly and axon growth (Nieuwenhuis et al., 2020). Our prior research employing network pharmacology and molecular docking indicates a potential association between the therapeutic efficacy of IRFV in SCI rats and the PI3K/Akt signaling pathway (Wang et al., 2023). *In vivo*, we found that the phosphorylation of Akt was upregulated in the spinal cord tissues of SCI rats treated with IRFV. Additionally, we employed the PC12 cell oxidative stimulation model to investigate the relationship between IRFV’s axonal regeneration effect and the PI3K/Akt signaling pathway. The findings indicated that, alongside axonal regeneration, there was an upregulation in the expression of PI3K/Akt signaling pathway proteins in PC12 cells treated with IRFV. Conversely, the axonal regeneration-promoting effect of IRFV was reversed in PC12 cells treated with LY294002, an inhibitor of the PI3K/Akt signaling pathway. Corresponding outcomes were similarly observed in *in vivo* experiments. In SCI rats administered intraperitoneally with LY294002, there was no improvement in pathological injury and motor function, nor was there an upregulation in NF200 and GAP43 protein expression; moreover, the axonal regeneration-promoting therapeutic effect of IRFV was counteracted. Collectively, these findings demonstrate that IRFV facilitates axonal regeneration and motor function recovery post-SCI by activating the PI3K/Akt signaling pathway.

The multifaceted composition of TCM herbs, characterized by their synergistic and multi-target effects, can compensate for the inherent limitations present in chemical drug applications (Lu et al., 2020). Employed as an adjunctive therapy, TCM has shown to be advantageous in enhancing motor and sensory functions, as well as the daily living capabilities of individuals with SCI (Zheng et al., 2020). VJJ, with its extensive historical application in TCM, exhibits a wide spectrum of biological activities, including sedative-hypnotic (Sah et al., 2010), antianxiety and antidepressant (Zhang B. et al., 2021; Zhang L. et al., 2021), anti-tumour properties (Tian et al., 2020). For CNS, studies have corroborated that VJJ modulates the hypothalamic–pituitary–adrenal axis (Yan et al., 2010; Shi et al., 2014), inhibit microglia activation (Yang et al., 2022), and protect the blood–brain barrier (Zhang B. et al., 2021; Zhang L. et al., 2021). Contrary to the findings of this study, VJJ has been previously demonstrated to inhibit rotavirus-induced diarrhoea by suppressing the PI3K/AKT signalling pathway (Zhang et al., 2021), and 11-Ethoxyviburtinal, derived from VJJ, exerts anxiolytic effects through this pathway’s inhibition (Lyu et al., 2023). These variations may stem from the distinct molecular mechanisms underlying various disorders and the diverse bioactive constituents present in VJJ.

The principal chemical components of VJJ comprise iridoids, volatile oils, flavonoids, among others (Jugran et al., 2019). Our prior network pharmacology research indicates that the predominant active constituents of VJJ effective in treating SCI are primarily iridoids (Wang et al., 2023). Iridoids, classified as monoterpenes, typically exist in forms such as cyclopentanes and pyrans, and are extensively found across diverse plant and animal species (Ndongwe et al., 2023). An iridoid compound isolated from *Hydrangea paniculata* has demonstrated neuroprotective effects in instances of PC12 cell injury (Shi et al., 2012). Cornus iridoid glycosides have been found to treat type 2 diabetes mellitus in conjunction with non-alcoholic fatty liver disease by activating the PI3K/Akt pathway in rats (Niu et al., 2021), while also exerting hypolipidemic, hypoglycemic, and antioxidant effects (Kang et al., 2018). Furthermore, they ameliorate cognitive impairment caused by chronic cerebral insufficiency (Wang et al., 2020), protect against ischemia-induced white matter lesions via the PI3K/Akt/mTOR pathway in the white matter (Wang J. et al., 2019; Wang M. et al., 2019), and facilitate SCI treatment by promoting axonal growth and extension into the lesion site (Tang et al., 2016). Catapol, an iridoid derivative isolated from Rehmannia glutinosa, has been shown to elevate GAP43 and synaptophysin levels in the hippocampus, thereby improving neuroplasticity loss (Liu et al., 2006). Additionally, it promotes axon regeneration in both *in vitro* and *in vivo* stroke models via the PI3K/AKT/mTOR pathway (Wang J. et al., 2019; Wang M. et al., 2019). Comparable outcomes were noted in the current study.

In conclusion, this study substantiates that IRFV facilitates axonal regeneration and motor function recovery post-SCI through the activation of the PI3K/Akt signaling pathway, thereby offering novel evidence supporting the application of VJJ in SCI treatment. However, this study is not without its limitations. Firstly, this experiment predominantly investigated the therapeutic effects of IRFV, a broad category of compounds, necessitating further exploration into the specific chemical constituents responsible for its pharmacological actions. Secondly, while this study confirmed the activation of the PI3K/Akt signaling pathway by IRFV, the mechanism of its *in vivo* target activation remains to be elucidated. This study lays a theoretical groundwork for the clinical translation of VJJ in SCI treatment and establishes a foundation for future explorations into SCI treatment strategies.

In future investigations, we will aim to delve deeper into the therapeutic efficacy of IRFV in SCI. Firstly, the experimental duration will be extended appropriately and multiple time points will be implemented to identify the optimal time point for observing IRFV-induced axon regeneration after SCI, and the effect of IRFV on the downstream PI3K/Akt pathway will be further explored. Additionally, Darts technique will be employed to further elucidate the constituents and targets of IRFV in SCI treatment. Secondly, we utilized *in vivo* neuron labeling technology or *in vivo* neuron imaging technology to delve deeper into the dynamic process of IRFV-induced axonal growth post-SCI. Secondly, *in vivo* neuron labeling technology or *in vivo* neuron imaging technology was used to further explore the dynamic process of IRFV-induced axonal growth after SCI. Thirdly, to investigate novel drug delivery approaches, we will explore the combination of IRFV with hydrogels, emerging nanomaterials, stem cells, and other agents to assess drug targeting efficiency and identify the optimal drug delivery method. Lastly, we will aim to integrate pertinent clinical trials to facilitate clinical application.

## 5 Conclusion

In this study, we employed a hybrid approach of both *in vivo* and *in vitro* experiments to initially explore the mechanism by which IRFV promotes axonal regeneration in SCI. Our findings indicate that IRFV targets and activates the PI3K/Akt signaling pathway, thereby facilitating axonal regeneration post-SCI, ameliorating spinal cord histopathological injury, and enhancing motor function recovery. In summary, this study potentially offers substantial theoretical support and a research foundation for the clinical application of IRFV, as well as for the subsequent drug development aimed at treating SCI.

## Data availability statement

The original contributions presented in the study are included in the article, further inquiries can be directed to the corresponding author.

## Ethics statement

The animal study was approved by the Ethics Committee of the General Hospital of the Western Theater Command of the Chinese People's Liberation Army. The study was conducted in accordance with the local legislation and institutional requirements.

## Author contributions

YW: Methodology, Software, Validation, Writing – original draft. JL: Investigation, Supervision, Writing – review & editing. HX: Software, Supervision, Writing – review & editing. LD: Resources, Supervision, Writing – review & editing. YH: Data curation, Supervision, Writing – review & editing. CC: Formal analysis, Methodology, Writing – review & editing. WW: Conceptualization, Writing – review & editing.
